# Understanding invisible pain in the older adult: a content analysis of social media’s representation of herpes zoster vaccine

**DOI:** 10.3389/fpubh.2024.1420349

**Published:** 2024-07-23

**Authors:** Mingyi Yang, Yiran Yang, Shuyue Li

**Affiliations:** School of Journalism and Communication, Guangxi University, Nanning, China

**Keywords:** HZV, social media, health information, the extended parallel process model, older adults

## Abstract

Herpes zoster (HZ), a common disease in older adults, affects their quality of life. Therefore, this study aimed to examine the blog posts of HZ-related information on social media platforms to analyze the attitudes and behaviors of residents toward the dissemination of health information. This research used content analysis to focus on Weibo, a representative social media in China, to analyze the content of 1866 blog posts related to herpes zoster (HZ) and herpes zoster vaccine (HZV). According to the consistency test by Cohen’s Kappa, four themes were identified: (a) sources, (b) tones, (c) epidemiological information, and (d) extended parallel process model elements. The findings showed that most information on Weibo came from non-professionals, with a neutral tone, and showed the invisible pain of HZ and the effectiveness of HZV through the two largest aspects of prevention and aged protection in epidemiological information. However, current blog posts treat the older adult as invisible individuals, failing to acknowledge them as recipients of the information. Additionally, the cost of the vaccine acts as an invisible economic barrier, contributing to the dissemination of incorrect information about folk remedies. This impacts the older adult’s acceptance of health information related to HZV. Thus, the way to share health information with the older adult needs to be improved in the future, and attention should be paid to the transmission of incorrect information to improve their vaccination rates and awareness of health management.

## 1 Introduction

Herpes zoster, also known as “Yaochanlong (Dragon’s Embrace)” in China, is an infectious skin disease that is usually accompanied by varying degrees of pain- or nerve-related symptoms (1). The disease does not directly affect life, but it can have a serious impact on the health-related quality of life of the patient. With an aging society, the incidence of HZ is increasing. Despite the significant impact of HZ, there is a considerable lack of awareness among the older adult regarding their susceptibility to this condition.

Social media, as an emerging and active platform for health information dissemination, allows users to post health-related information at any time and has a powerful influence on health decisions (2). At the same time, professional doctors and medical institutions have changed from single offline dissemination of health information to online and offline dual-channel output of professional health knowledge (3). In this new environment, Weibo is increasingly becoming a platform for people to obtain health information and exchange medical experiences in China (4). Chen et al. (5) pointed out that the younger generation sought information about the HZV through Internet searches and forwarded it to their parents. Conversely, the older generation preferred receiving more information about HZV directly from doctors. However, it is worth noting that the research on HZ and HZV is limited, largely focusing on their clinical manifestations and prevalence, with little focus on their presentation on social media, thus making it feasible to conduct research on HZ and HZV through social media platforms.

Therefore, it is important to understand how HZ and the HZV are presented on social media, and how it influences vaccination decisions. This study aimed to investigate the dissemination of health information on social media platforms and its impact on the public. Specifically, this paper conducts a content analysis of blog posts related to HZ and HZV on the Weibo platform in China. After coding the content, the thematic consistency is assessed using Cohen’s kappa, thereby gaining an in-depth understanding of how these blog posts present HZ and HZV, portray the older adult, and reflect the attitudes and perceptions toward health information from the public’s perspective. By analyzing these blog posts, the study assesses the potential impact of health information on Weibo on the health decisions and perceptions of the older adult population, as well as its possible influence on their attitudes and behaviors toward HZV vaccination. In this way, the paper hopes to improve health information dissemination strategies on social media, providing a scientific basis for enhancing public awareness and acceptance of HZV vaccination.

Accordingly, this study addressed the following research questions:

*RQ1.* How does Weibo present health information related to HZ and HZV?

*RQ2.* How does Weibo portray the older adult in these blog posts?

*RQ3.* What attitudes and perceptions do EPPM items in blog posts reflect about people’s views toward the HZV?

## 2 Literature review

### 2.1 Herpes zoster and herpes zoster vaccine

Herpes zoster (HZ), a prevalent and debilitating disease characterized by painful vesicular rash (6), poses a significant health threat to the older adult population worldwide. In the context of rapid aging in the Asia-Pacific region, the average incidence of HZ is estimated to be 3–10 per 1,000 person-years. HZ incidence rises steeply above 40 years and peaks at 70–80 years (7). Approximately 5–30% of HZ patients will experience postherpetic neuralgia, a disease sequelae characterized by persistent pain after rash remission, lasting at least 3 months (8). Currently, the herpes zoster vaccine (HZV) is the only means of primary preventing HZ and is the most effective and feasible preventive measure (7–11).

Despite the importance of HZV in preventing HZ, public willingness and acceptance of the HZV remain low (5). The willingness of older adult populations aged 50 years and above in various World Health Organization (WHO) regions to receive herpes zoster vaccines did not exceed 50%. In particular, the willingness rates varied across regions: the Eastern Mediterranean region showed a willingness rate exceeding 70%, while the Western Pacific region’s rate was approximately 55%. In East Asia, China was among the countries with the lowest willingness rate (12). The lack of awareness of HZV may contribute to the low willingness rate in China. Research has found that 79.7% of respondents in Xi’an, China, are unaware of HZV (13). Therefore, it is critical to increase awareness to enhance willingness to vaccinate for HZV. First, perceived barriers to vaccination have been found to be the main reason for public unwillingness to vaccinate (14). Vaccination availability, disease complications (15), and trust in the effectiveness of the HZV are also contributing factors to HZV vaccination rates. Moreover, advice from healthcare professionals plays an important role in enhancing public willingness (16). Thus, it is meaningful to regard HZ and HZV information on social media as objects of study.

### 2.2 Online health information and seeking behavior for the older adult

Media is the main platform for health communication, allowing the public to better understand health information, thus enabling health behaviors, cognitive attitudes, and behavioral change (17). With the popularization of the use of the Internet, social media has gradually become a media channel for residents to receive health information.

Online media has significantly influenced public perceptions of vaccines (18). However, few studies have examined the HZV-related health information in social media. Therefore, taking other preventive vaccinations is necessary. Human papillomavirus (HPV) is also preventable through vaccination; previous research on HPV-related information in social media has shown that social media plays a pivotal role as an important source of HPV-related information (19). Research studies have demonstrated that engagement with HPV vaccination information through online searches, discussions, and shared decision-making processes positively influences vaccine knowledge, attitudes, and intentions (20). In addition, social media promotion has been found to reduce perceived risk and increase Chinese parents’ support for their children’s HPV vaccination decisions (21).

Although many older adult people have begun to use the Internet or social media to access health information (22), this percentage is still relatively small compared to the large Chinese older adult population. However, research suggests that adult children may seek online health information for their aged parents. Specifically, adult children in China have a more positive attitude when searching for health information for their older adult parents and take on the responsibility of caring for the health of their older adult parents and feel obligated to search for health information on their behalf (23, 24). Moreover, the perceived severity of the aged parents’ illness could motivate adult children’s online health-seeking behavior (25). However, Koskinen et al. (26) have pointed out that such behavior may overlook the autonomy of the older adult and may imply paternalism or ageism. Therefore, studying the spread of health information related to HZ and HZV on social media holds significant value.

### 2.3 Portrayals of older adults in the media

Social media is becoming increasingly influential, which has empowered individuals to spread information by themselves. In particular, more and more older adult people are actively using social media for online information searches and self-representation (27).

However, previous studies have shown contradictory results concerning the role of social media in reinforcing or challenging the stereotypes of the older adult. On the one hand, Twitter appeared to be a platform for reproducing and reinforcing discussions about older adults as a disempowered, vulnerable, and homogenous group (28). In particular, during the prevalence of COVID-19, negative tweets about COVID-19 and aging often characterized older adults as helpless and expendable individuals (29). Similarly, Facebook was also considered to mainly convey the negative stereotypes of the older adult group (30). On the other hand, YouTube videos under the labels of *“old age”* or *“senior citizens”* have challenged the negative stereotypes mentioned above (31). These contradictory results prove that the media portrayals of old people might be different on different social media platforms. Hence, it is necessary to analyze how the older adult population is represented in related content on Weibo.

### 2.4 The extended parallel process model

Fear is one of the basic human emotions (32). In health communication, fear appeal is a common persuasive strategy that promotes self-protective behaviors by showing the public the negative consequences of not practicing health behaviors and eliciting their fear (33). Based on it, Witte (34) developed the extended parallel process model (EPPM). This model suggests that a fear appeal message will trigger the audience to first engage in a threat-level assessment process, which the audience will measure in terms of two dimensions: perceived severity and perceived susceptibility. Then, it combines the fear control process and the danger control process.

In studies applying the EPPM, the results of most studies validate its value in predicting the effectiveness of fear appeal. McKay et al. (35) used the EPPM as a framework for designing health messages and testing the adherence of older adults, and the results of the experiment showed that older adults who did not take the advice originally were more confident in their ability to do so after reading the high-threat/high-performance messages. Jin et al. (36) have explored the relationship between fear appeals and HIV prevention behavioral interventions and have affirmed the significance of fear appeal messages in reducing high-risk HIV behaviors. Therefore, the third research question is proposed as follows:

Moreover, upon receiving an others-oriented threat message, there is a willingness to adopt the behavioral recommendations associated with the message to protect others. Overall, fear appeal messages had equally significant effects when directed threateningly toward others rather than themselves. Most threat messages are more biased or default to the threat being a self-oriented threat, i.e., the threat is of the self rather than of others. It has been shown that describing to smokers the threat that smoking poses to their family’s health is more persuasive than routinely describing the threat to themselves (37). In addition, when recommending behavior suggestions to a group, emphasizing the protection of their family and group may be more useful for social groups that advocate collectivism (38). Compared to other countries, China emphasizes collectivism more. A study found that Chinese audiences are more concerned with collective interests than individual interests (39). The health threat of herpes zoster goes beyond the level of the susceptible population and causes worry in the generation of children in the family as well. Consequently, conducting information analysis from the perspective of the extended parallel process model (EPPM) is feasible.

## 3 Method

### 3.1 Sampling

Weibo, as a representative social media platform with comprehensive information in China, boasts approximately 588 million monthly active users and is increasingly recognized as an important channel for representing health-related information. This platform offers a publicly available application programming interface (API), facilitating the retrieval of Weibo’s public data. For the purposes of this study, we utilized “Octoparse,” a third-party Python software, to extract Weibo posts within the timeframe extending from May 1, 2023 to November 30, 2023. The reason for choosing this timeframe is that the domestically produced herpes zoster vaccine in China officially began to be administered. The dataset amassed encompassed various data fields and content details, encompassing the posts’ content, authors, URLs, timestamps of posting and updating, and metrics on likes, comments, and shares.

**Figure 1:**
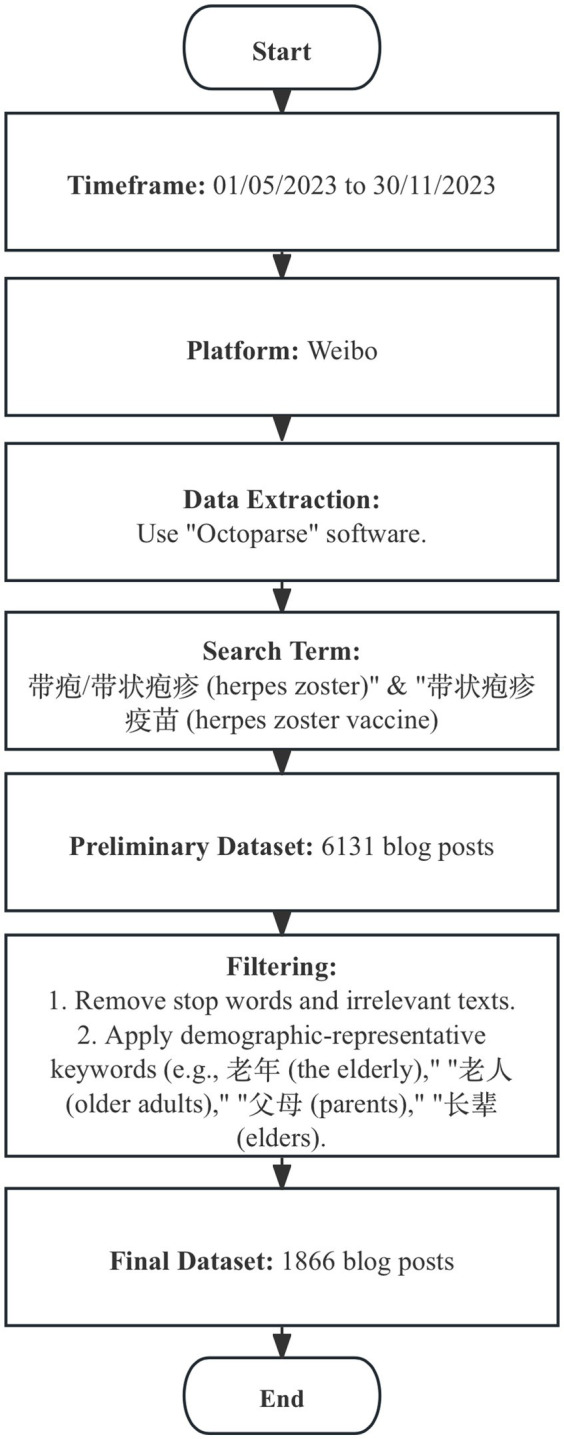
Flowchart of blog post selection.

Employing specific search terms relevant to herpes zoster, including “带疱/带状疱疹(herpes zoster)” and “带状疱疹疫苗 (herpes zoster vaccine),” this study obtained a corpus of 6,131 posts. Subsequently, this study utilizes the Harbin Institute of Technology (HIT) stopword database to exclude commonly occurring, non-informative terms in Chinese such as “和(and),” “不仅(not only),” and “一个(a).” Then, it filters with demographic-representative keywords such as “老年(the older adult),” “老人(older adults),” “父母(parents),” and “长辈(elders).” Finally, 1866 blog posts were extracted (Figure 1).

### 3.2 Coding procedure

The coders, two postgraduate students specializing in journalism and communication, were tasked with achieving comprehensive familiarity with the data by engaging in thorough reading and re-reading to gain a deep understanding of its content. Subsequently, they generated codes to highlight significant features within the data. These codes were analyzed to identify essential patterns and generate initial themes. Then, these potential themes were examined against the data to confirm they represented the content accurately, providing comprehensive explanations and instances for each coding term. It is important to note that in the process of coding, some blog posts covered multiple types of epidemiological information, leading to the same information being categorized into different types. To ensure a consistent approach and address any differences in the coding scheme, regular meetings were organized among the team members. This study constructed themes and subthemes to emphasize older adults as well as social media as a platform. Specifically, they are (a) information source: official media, medical institution, Internet-based media, public institution, professional, and non-professional; (b) tone: positive, neutral, and negative; (c) presence of epidemiological information: prevention of cancer, recommended age, risk of the vaccine, side effects, and follow-up test after vaccination; (d) presence of EPPM terms susceptibility, severity, self-efficacy, and response efficacy.

The purpose of the consistency test for content analysis is to pursue the stability and accuracy of the category constructs. Therefore, after pre-coding was completed, 165 blog posts (8.84% of the sample) were systematically sampled as materials for the reliability test. Cohen’s Kappa was used for the consistency test, during which coders began checking for inter-participant reliability. The average reliability of the coders who counted all the items was calculated to be 0.89. The resulting coefficients indicated that the agreement between the two coders (Cohen’s Kappa >0.8) was at a high level and met the criteria for agreement in content analysis coding studies. For detailed information on interrater reliability, definitions of themes, and examples of posts, refer to Table 1.

**Table 1:** Coding scheme.

Theme and subtheme		Interrater reliability (Cohen’s kappa)	Definition	Example
Information source	Official media	0.827	Accounts of the media that are owned by governments and are allowed to conduct news interviews in China	Guangming Daily, CCTV news
	Medical institution	0.814	Accounts of organizations that provide medical services to the public	Hospitals, medical service centers
	Public institution	0.904	Accounts of non-profit organizations that serve for public’s interests	Government agencies, educational institutions, public service organizations
	Internet-based media	0.869	Accounts of online news media that are allowed to conduct news editing but not allowed to conduct news interviews	Toutiao (Chinese news and information content platform)
	Professional	0.938	Accounts of non-anonymous persons who are from medical fields and have medical training experiences	Doctors, nurses, medical experts
	Non-professional	0.902	Accounts of persons who do not have professional medical training experiences	Celebrities, influencers, intellectuals
Tone	Positive	0.948	Attitudes toward the uptake of the herpes zoster vaccine	The herpes zoster vaccine introduced in China this time is the most advanced in the world, with a protection rate of over 97%.
	Neutral	0.915		There are many benefits to getting the zoster vaccine, but the price of 3,000 yuan for two doses has really deterred most parents.
	Negative	0.886		I used to get this illness every time summer turned to autumn when I was a child, and it was cured with folk remedies. There’s no need for vaccination.
The Presence of Epidemiological Information	Prevention of herpes zoster	0.884	Information about the effectiveness of HPV vaccines in preventing cancers	Getting vaccinated against herpes zoster not only effectively reduces the incidence of herpes zoster and lessens the severity of the disease but also decreases the rate of postherpetic neuralgia, a complication of herpes zoster.
	Age protection	0.951	Information about the age groups potentially affected by herpes zoster	The older adult, women, people with low or compromised immunity, and individuals who have recently been overfatigued or under great stress are at high risk for herpes zoster.
	Recommended age	0.977	Information about the age group to be recommended for zoster vaccination	It is recommended that people over the age of 50 years get vaccinated against shingles as soon as possible.
	Risk of vaccine	0.941	Information about the long-term consequences and uncontrollability of zoster vaccines	Individuals allergic to the vaccine components, women who are breastfeeding, and people who are in the active phase of herpes zoster should not be vaccinated for the time being.
	Side effects	0.821	Information about the immediate reactions after the injection	Some people may experience symptoms such as pain at the injection site, muscle aches, headache, and chills after receiving the zoster vaccine.
	Follow-up test after vaccination	0.920	Information about regular testing after zoster vaccination	After receiving the herpes zoster vaccine, follow-up tests are required.
The Presence of EPPM Terms	Susceptibility	0.925	Information about the people who are more likely to be infected with herpes zoster	People over the age of 50 years are at high risk for herpes zoster.
	Severity	0.895	Information about the consequences of zoster infection (e.g., specific symptoms)	Wang used to be healthy, but after contracting herpes zoster, he suffered from severe neuralgia. Sleepless nights led him to several bouts of suicidal thoughts.
	Self-efficacy	0.834	Information about the measures and guidance for preventing zoster infection (e.g., place and cost of zoster vaccine uptake)	In Wuhan, people aged 50 years and above can go to community health service centers to receive the zoster vaccine by making an appointment.
	Response efficacy	0.890	Information about the effectiveness of zoster vaccines in preventing herpes zoster	Vaccination is an economical, effective, and simple means of disease prevention that can help the older adult reduce the threat of illness and effectively lower the risk of severe complications and mortality.

## 4 Results

### 4.1 Sources and tone of herpes zoster information

Sources. This study extracted six subthemes from the dataset. The main sources of blog posts about HZ and HZV were individuals (non-professional: *n* = 802, 43.0%), followed by public institutions (*n* = 395, 21.21%) and official media (*n* = 294, 15.76%). This finding is similar to the study by Yang et al. (40) on tweets related to ticks and tick-borne diseases. Additionally, this analysis identified Internet-based media (*n* = 135, 7.27%) and professional media (*n* = 192, 10.30%) as an important part of courses. However, medical institutions were the least likely source of information on HZ and HZV.

Tone. Three subthemes were identified for this theme: positive, neutral, and negative. The findings revealed that blog posts with positive tone (*n* = 238, 12.73%) mainly highlighted the vaccine’s advanced nature and its high efficacy in protection against herpes zoster. On the other hand, blog posts with a negative tone (*n* = 266, 12.21%) frequently expressed doubts or concerns about the necessity of HZV. Furthermore, it was noted that individuals tended to share personal or older adults’ disease experiences with HZ, mostly neutral (*n* = 1,402, 75.15%).

### 4.2 Epidemiological information

This study categorizes epidemiological information of health information related to HZ and HZV into six types: prevention of herpes zoster (*n* = 1,380, 44.53%), age protection (*n* = 1,018, 32.85%), recommended age (*n* = 294, 9.49%), risks of vaccine (*n* = 226, 7.29%), follow-up testing after vaccination (*n* = 68, 2.19%), and side effects (*n* = 113, 3.65%).

Approximately half of the blog posts provide information on the prevention of herpes zoster, indicating that most posts tend to describe the preventability of HZ, such as effective prevention through vaccination and boosting immunity. In addition, a third of the information is related to the theme of age protection, indicating that most blog posts emphasize the older adult population, such as their higher susceptibility to HZ, and encourage their children to take action (“We recommend children take parents to get the HZV as soon as possible”). However, there is less description related to the recommended age and the risks of the vaccine, with only a few posts suggesting that the best age for HZV vaccination is over 50 years for older adult people and that vaccination against HZV may impact chronic diseases. The least amount of content is on follow-up testing after vaccination, with only a few people sharing their post-vaccination experiences, including asymptomatic cases and potential pain scenarios.

### 4.3 Characteristics by EPPM elements

This study organized the blog posts into four subthemes according to the components of the extended parallel process model (EPPM), including perceived susceptibility (*n* = 1,131, 25.85%), perceived severity (*n* = 1,244, 28.43%), self-efficacy (*n* = 1,255, 28.68%), and response efficacy (*n* = 746, 17.05%).

Overall, the four subthemes are relatively evenly distributed in health information related to HZ and HZV. The subtheme of high susceptibility focused on the closeness of HZ to the older adult population and discussed the substantial number of older adult individuals affected by HZ, its prevalence in China, and the elevated risk of HZ among older adults. In addition, the high-severity subtheme aimed to influence the audience’s perception of the disease’s seriousness through descriptions of physical discomfort and pain (e.g., “pain in pain”), psychological distress (e.g., “dark mood”), and various adverse outcomes associated with HZ, such as insomnia, loss of appetite, disease recurrence, and neurological complications.

Furthermore, the subtheme about high self-efficacy included posts that stressed the importance of good health habits to avoid HZ, especially pointing out how crucial it is to get the HZV and to go to a doctor for professional advice. On the other hand, a notable number of blog posts exhibited low response efficacy, disseminating inaccurate health information that questioned the effectiveness of HZV vaccination with claims like “you will still get sick after inoculation” and promoting the misconception that HZ can be easily treated post-infection.

## 5 Discussion

### 5.1 Invisible pain and concerned HZ-related health information

From the analysis of sources, tone, and epidemiological information, this article finds that Weibo effectively presents health information related to HZ and HZV. Regarding the source of information, official media, Internet-based media, and professionals emphasized the specific group of older adults, aiming to provide health-related information to susceptible populations. Epidemiological information of posts presented the severity, susceptibility, and potential side effects of herpes zoster, while also emphasizing the efficacy of HZV. As to details, most of the articles focused on the prevention of herpes zoster, suggesting that the disease can be prevented by vaccination, which is in line with the current level of treatment for the disease. However, the follow-up testing after vaccination and side effects of the vaccine are rarely mentioned, which fails to inform of the risks. It may be associated with the fact that the HZV has just begun to be administered in China.

Utilizing social media platforms such as Weibo to share health information about HZ and HZV assists Chinese residents in gaining a deeper understanding of it. Worldwide, the willingness of older adult populations aged 50 years and above in various World Health Organization (WHO) regions to receive herpes zoster vaccines (HZVs) did not exceed 50%. In particular, the willingness rates varied across regions: the Eastern Mediterranean region showed a willingness rate exceeding 70%, while the Western Pacific region’s rate was approximately 55%. China was among the countries or regions with the lowest willingness rate (14). Increasing health information dissemination and broadening knowledge accessibility can alleviate vaccine hesitancy among the public, heighten disease prevention awareness, and foster a willingness to get vaccinated as well as actual vaccination actions.

In summary, blog posts have delivered the dissemination of health information rather than fear appeal, promoting individuals to take health actions, such as accompanying their parents for HZV vaccination.

### 5.2 Invisible older adults and their absence on social media

The older adult, as a susceptible group to HZ, are supposed to be the main target for the communication of health information related to HZ. However, the HZ-related blog posts on social media often emphasize connections with offspring while overlooking the subjective being of the older adult themselves. Although people care about the older adult’s longevity of life, there is a notable indifference toward their health-related quality of life.

On one hand, children’s awareness of HZ or HZV information on Weibo may directly influence parental perceptions and vaccination. Currently, the information society has led to a cultural feedback revolution within families, with new generations often helping older generations to access online information (41). Therefore, when scholars conducted studies on HZ and HZV in the Asia-Pacific region, the group of adults whose parents were older than 50 years were also included in the interviews, and they were considered to be the key decision makers on whether their parents received vaccination and healthcare coverage (5). The findings of this paper support the notion suggested by Antonopoulos et al. (42), which indicated the existence of a third-person effect in attitudes toward information on media websites.

On the other hand, while Weibo become an important channel for disseminating HZ or HZV information, the current information dissemination has overlooked the physical and psychological abilities of the older adult and has not fully reflected the needs of the older adult as independent individuals. These posts about HZ and HZV are similar to past portrayals of the older adult in traditional media, as a group unable to access health information; that is, they have a negative attitude toward health knowledge on the Internet and have low digital literacy. Social media still seems to reinforce the ageist discourse that exists in traditional media (28). This coincides with the research results of this article; that is, even though the older adult group is susceptible to HZ and needs to be vaccinated with HZV, they are forced to be absent on social media.

In general, the health information related to HZ and HZV presented on Weibo neglected older adults as a person with physical and mental capacities and showed that they are affiliated with the younger generation.

### 5.3 Invisible economic burdens and misunderstanding related to vaccination

The current investigation highlights a trend: while there is widespread concern for HZ-related health information, there is misinformation and misunderstanding about its preventive and treatment modalities in terms of health information.

The high price of HZV has become an invisible economic barrier to the older adult population. From one perspective, the high susceptibility and severity associated with HZ and HZV in the blog posts effectively facilitated the acceptance of health information. These elements play an important role in influencing individuals’ perceptions and motivating them to consider preventive actions. The portrayal of high susceptibility and severity in the blog posts emphasizes the susceptibility of HZ to the older adult population and the significant discomfort and potential complications it can cause. This representation likely heightened readers’ perceived threat of the disease, aligning with the statement of EPPM that a higher perceived threat can lead to greater engagement with health messages.

However, despite the effective communication of preventive measures toward HZ, the high cost of the HZV emerged as an invisible barrier. Currently, the price of domestic HZV in China is approximately US $214 (43). However, the price provided by the CDC (44) is US $120.9 per dose, and participants in medical insurance programs may not have to pay more than US $50 (45). Further examining the income levels of residents in both countries reveals that China’s *per capita* disposable income stood at merely US $6,033 in 2023 (46), significantly lower than the average personal income of US $68,531 in the USA (47). Considering the payment ability of most Chinese residents, the economic burden of the HZV is comparatively heavy, significantly affecting its affordability. This economic burden significantly influences the dissemination of HZV health information and the subsequent action it aims to inspire. The invisible discrepancy between the willingness to act based on perceived threat and the ability to act due to financial constraints shows a critical gap in public health communication.

In addition, this article finds that high self-efficacy and low response efficacy have facilitated the spread of incorrect information about HZ and HZV, thereby disrupting the accurate communication of health information. High self-efficacy in blog posts, which reflects individuals’ confidence in their ability to perform specific actions to prevent or alleviate health issues, emphasizes proactive health behaviors in discussions, such as vaccinations. However, this positive aspect was counteracted by low response efficacy, where individuals doubted the effectiveness of these actions, particularly the efficacy of the HZ vaccine, and a belief in prevention or post-disease treatment by folk remedies.

In the topic which was named #We recommend children take parents to get the HZV as soon as possible#, in contrast to older adults, who were identified as the target population for HZV, it seems that younger adults’ responsibility for paying their families for vaccinations was emphasized in social media. In a collectivist country, health communication is more effective through concern for the health of family members. However, as the HZV is not covered by health insurance in China, the price of this local production vaccination is still high, and young adults find it difficult to afford, stating that they “do not know why it is sold at such a high price.” Meanwhile, it is of concern that some young adults are arguing that HZ does not need to be prevented and mentioned “there are folk remedies to treat it and there is no need to spend a lot of money to vaccinate your parents.” These folk remedies, which are recommended by non-identified medical accounts, are spread around Weibo under the guise of Chinese traditional medicine. They kept the opinion that vaccination can cause an economic burden. However, in fact, the cost of vaccines formed without vaccination is higher (48).

However, it is of concern that there are still some blog posts that argue that the disease does not need to be prevented and that “there are native remedies to treat it and there is no need to spend a lot of money to vaccinate your parents.” From the analysis of the EPPM items toward HZ-related health information, the high number of “low response efficacy” posts on Weibo is of concern. It contains misinformation about the ineffectiveness of HZV vaccination and the possibility of treating the disease after it has occurred, and there are also low-severity presentations of information, which presented the belief that HZ is not serious, downplaying the sequelae such as PHN. This is related to the dissemination of individuals who do not have specialized knowledge and experience in the medical field, and the information provided by non-experts can cause people to act based on unsubstantiated personal experience and to be skeptical about the accuracy and credibility of the health information provided by professionals. In conclusion, while blog posts have successfully communicated the risks associated with HZ and the importance of vaccination, the high cost of the HZV presents a significant economic barrier, particularly for the older adult population. Furthermore, the combination of high self-efficacy and low response efficacy has led to the spread of incorrect information about HZ and HZV, complicating the environment of health communication.

## 6 Conclusion and limitation

Perception of HZ disease affects HZV vaccination rates (49), and existing health information has increased residents’ awareness of HZ and aroused their attention to vaccination. This study concludes that although platforms such as Weibo are important in spreading health information related to herpes zoster vaccine (HZV), the prevailing discourse inadequately caters to the older adult demographic in China. This study concludes that although platforms such as Weibo play a significant role in disseminating health information related to HZ and HZV, the prevalent discourse does not adequately meet the needs of the older adult population. Furthermore, the spread of incorrect information has influenced the spread of accurate health information and reduced the awareness and vaccination rate of HZV in the older adult group. To more effectively meet the needs of the older adult population and curb the spread of misinformation, it is crucial to enhance the accessibility and comprehensibility of health information, strengthen health education and public awareness, and provide financial support and incentives.

There are some limitations in this study. First, although all the keywords related to HZ have been searched, the resulting sample size is still insufficient; most of the data collection was conducted through the Internet, and there is a certain amount of information bias. The present study was conducted to understand the cognitive presentation of HZ in social media content through posts to provide a factual basis for future publicity and vaccination strategies for HZV. To reduce the impact of HZ on the quality of health and life of the older adult population, further data collection on multiple platforms is needed in the future to explore specific measures to improve people’s awareness of HZ and to promote healthy aging under the reality of the gradual population aging.

## Data availability statement

The original contributions presented in the study are included in the article/supplementary material, further inquiries can be directed to the corresponding author.

## Author contributions

MY: Conceptualization, Data curation, Formal analysis, Funding acquisition, Investigation, Supervision, Writing – original draft, Writing – review & editing. YY: Conceptualization, Data curation, Formal analysis, Funding acquisition, Investigation, Methodology, Project administration, Resources, Software, Supervision, Validation, Visualization, Writing – original draft, Writing – review & editing. SL: Data curation, Methodology, Validation, Writing – review & editing.
